# Swallow-related quality of life and oropharyngeal dysphagia in myotonic dystrophy

**DOI:** 10.1007/s00405-020-05964-2

**Published:** 2020-04-15

**Authors:** Walmari Pilz, Valéria Lima Passos, Rob J. Verdonschot, Jolanda Meijers, Nel Roodenburg, Yana Halmans, Catharina G. Faber, Bernd Kremer, Laura W. J. Baijens

**Affiliations:** 1 Department of Otorhinolaryngology, Head and Neck Surgery, Maastricht University Medical Center, P.O. Box 5800, 6202 AZ Maastricht, The Netherlands; 2 School for Mental Health and Neuroscience, MHeNs, Maastricht University, Maastricht, The Netherlands; 3 Department of Methodology and Statistics, Maastricht University, Maastricht, The Netherlands; 4 School for Public Health and Primary Care, CAPHRI, Maastricht University, Maastricht, The Netherlands; 5 Emergency Department, Erasmus Medical Center, Rotterdam, The Netherlands; 6 Department of Radiology, Maastricht University Medical Center, Maastricht, The Netherlands; 7 Department of Neurology, Maastricht University Medical Center, Maastricht, The Netherlands; 8 School for Oncology and Developmental Biology, GROW, Maastricht University, Maastricht, The Netherlands

**Keywords:** Deglutition disorders, Dysphagia, Myotonic dystrophy, Quality of life, Videofluoroscopy

## Abstract

**Purpose:**

This study describes swallow-related quality of life (SWAL-QOL) in patients with myotonic dystrophy type 1 (DM1) and investigates its association with swallowing function and disease severity.

**Methods:**

A SWAL-QOL questionnaire was completed by 75 DM1 patients and 25 healthy control subjects. The severity of the disease was evaluated using the muscular impairment rating scale (MIRS). Twenty-eight DM1 patients underwent a videofluroscopic swallowing examination (VFS). Spearman’s correlation coefficient was used to measure the direction and strength of associations.

**Results:**

The SWAL-QOL median scores were significantly lower for the DM1 group than for the healthy control group. The scores for the majority of the SWAL-QOL domains were lower in patients with proximal muscular weakness (MIRS 4 and 5). Postswallow vallecular pooling and piecemeal deglutition were the most impaired VFS outcome variables.

**Conclusion:**

Our results suggest that a multidimensional swallowing assessment is recommended for DM1 patients as SWAL-QOL and VFS measure different aspects of the swallowing function, thus providing complementary information.

## Introduction

Myotonic dystrophy type 1 (DM1) is the most common form of muscular dystrophy in adults and is dominantly inherited. The clinical manifestations, which are highly variable, include myotonia, muscular dystrophy, cataracts, and involvement of other organs such as the heart, endocrine system, and brain. In addition, cognitive dysfunction, reduced initiative, inactivity, and apathy are consistent with the disease, and these characteristics have healthcare implications for DM1 patients [1]. Fatigue and reduced mobility have been identified as the symptoms most disturbing to their daily life [2]. Moreover, since the weakness present in many parts of the body also affects oropharyngeal muscles, swallowing impairment is prevalent in DM1 [3, 4].

DM1 patients report lower QoL than healthy subjects and these scores were associated to fatigue, daytime sleepiness, and muscular impairment [5, 6]. Although these prior studies have evaluated the influence of its physical and psychological manifestations on health-related quality of life (QoL), little is known about the impact of swallowing impairment on health-related QoL in DM1 patients. Besides fulfilling a basic need, eating has significant psychological and social functions. An evaluation of swallowing should therefore cover not only its physiological aspects but should also take patients' perception into account. To that end, data would have to be gathered on the impact of swallowing impairment on health-related QoL. A better understanding of how DM1 patients perceive and cope with oropharyngeal dysphagia could improve the guidelines for clinical interventions and rehabilitation programs. From that perspective, the aim of this study was twofold: (a) to describe swallow-related QoL in dysphagic patients with DM1, and (b) to investigate the relationship between swallow-related QoL with swallowing function on the one hand and disease severity on the other hand.

## Material and methods

### Participants

Genetically confirmed DM1 patients were prospectively included in this study and were consecutively recruited from the multidisciplinary outpatient clinic for dysphagia in the Maastricht University Medical Center (MUMC). All recruited patients were referred for swallowing examination on the grounds of signs or symptoms of deglutition disorders, i.e., cough while eating, sensation of food getting stuck in the throat, long time to finish a meal, etc. Individuals were excluded if they had any other neurological disease (besides DM1) or head and neck cancer; had received speech therapy in the past (to exclude benefit of treatment and attention); had cognitive impairment [mini mental state examination (MMSE) < 23]; or had undergone surgery of critical structures involved in swallowing (tongue, larynx, etc.) or the central nervous system. Patients over eighty years old were excluded due to the possibility of presenting dysphagia as a result of aging (presbyphagia). Healthy participants without swallowing complaints were recruited from the local community to serve as a control group. Informed consent was obtained from all participants. The study protocol was approved by the medical ethics committee of the MUMC.

### Measures

#### Disease severity

The neuromuscular involvement of the disease was scored by a neurologist using the muscular impairment rating scale (MIRS) [7]. This scale expresses a clinical assessment of the progression of muscular impairment (distal to proximal) in DM1. It classifies the degree of impairment in an ordinal scale ranging from one (no muscular impairment) to five (severe proximal weakness).

### Swallow-related QoL (SWAL-QOL)

The SWAL-QOL survey was designed to evaluate the impact of swallowing problems on the health-related QoL of patients with oropharyngeal dysphagia [8]. The questionnaire was translated into the Dutch language and then validated [9]. The SWAL-QOL was designed to assess eight domains of swallow-related QoL (general burden, food selection, eating duration, eating desire, fear of eating, communication, social functioning, and mental health), two concepts of generic QoL (fatigue and sleep), and a dysphagia clinical symptom scale (symptom score). The score of each domain is calculated based on two or more questions. The score per domain ranges from 0 (extremely impaired) to 100 (no impairment).

### Swallowing function

Swallowing function was evaluated by performing a videofluoroscopic swallowing study (VFS). During the VFS, the patients were offered one trial of thin liquid (low-density barium—40% w/v(weight/volume) and one trial of thick liquid (50 cc applesauce + 150 gr barium powder) followed by one bite-sized cracker coated with barium paste. All measurements followed the same protocol [10]. Each participant swallowed the bolus consistencies upon command and in the same sequence (thin liquid, thick liquid, and bite-sized cracker). The VFS was performed in a lateral position. The images were obtained with a Philips Diagnost 97 system (Philips Medical Systems, Eindhoven, The Netherlands) and recorded on DVD at 30 frames per second. All swallows were analyzed by two experienced raters trained in the VFS scoring system. They scored four ordinal variables: piecemeal deglutition (sequential swallowing on the same bolus), postswallow vallecular and/or pyriform pooling (bolus retention in the valleculae or pyriform sinus after swallowing), laryngeal penetration (bolus in the laryngeal vestibule above or on the level of the vocal folds) and aspiration (bolus passes below the vocal folds) [4]. The raters were blinded to each other’s results (independent rating) and to the patients’ medical history. A written manual with well-defined descriptions of the scales’ levels was available during the rating process. The swallows were scored in randomized order at varying speed (slow motion, normal, up to frame-by-frame speed) using the software program Windows Movie Maker version 5.1 (Microsoft Corporation, Redmond, WA, USA). The raters were advised to limit the duration of the measurement sessions (max. two hours) to avoid fatigue. To obtain intrarater agreement, each rater repeated the measurement of 39 swallows within a period of two weeks. The scores of the rater with the highest indices of intrarater agreement were used for subsequent statistical analysis.

### Statistical analysis

Raters’ agreement was analyzed with a weighted Kappa (for ordinal variables). The internal consistency of the SWAL-QOL was analyzed using Cronbach’s alpha. The participants' characteristics are presented as absolute numbers/percentages for categorical variables and median and 25th; 75th percentiles for continuous variables. Spearman’s correlation was used to measure the direction and strength of correlations. The two groups (DM1 patients and healthy control subjects) were compared with the Mann–Whitney *U* test. The internal consistency of the SWAL-QOL was analyzed using Cronbach’s alpha. Significance level was 5%. Because of the large number of null-hypothesis tests conducted, especially the many pair-wise correlations, *p* values were adjusted for multiple-testing with False Discovery Rate (FDR) correction.

## Results

### Characteristics of the participants

SWAL-QOL was completed by 25 healthy control subjects and 75 DM1 patients. The mean age in the control group was 45.6 years, ranging from 22 to 71, and 52% were men. In the patient group, the mean age was 43.43, ranging from 21 to 73, and 55% were men. Most DM1 patients presented mild to moderate proximal muscular weakness (MIRS 4, *N* = 22) or severe proximal muscular weakness (MIRS 5, *N* = 23). Eight patients presented minimal signs of muscular impairment (MIRS 2).

All healthy control subjects and over 68% of the patients completed the questionnaire by themselves. The other 32% of the patients received help, mainly with reading the questions and/or writing the answers. Most of the patients reported having a normal diet without texture modification.

### SWAL-QOL

The scores of all SWAL-QOL domains were significantly lower for the DM1 group than for the healthy control group (Table 1). In the healthy control group most of the SWAL-QOL domains showed a skewed distribution with an accentuated ceiling effect. No floor effect was observed. In the patients’ group, three SWAL-QOL domains presented a higher percentage of ceiling effect: ‘social functioning’ (56%), ‘eating desire’ (48%), and ‘fear of eating’ (48%). The dysphagia symptoms most frequently reported in the study were: ‘coughing’, ‘having to clear the throat’, and ‘food sticking in the throat’. No correlations were found between age, gender, and each of the eleven SWAL-QOL domains.

**Table 1:** Differences on SWAL-QOL domains between DM1 patients and healthy subjects

SWAL-QOL domains	DM1 patients *N* = 75	Healthy control subjects *N* = 25	*p* value^a^	Cronbach’s *α*^b^
Burden	88 (63; 100)^c^	100 (100; 100)^c^	0.001	0.90
Food selection	75 (50; 100)	100 (100; 100)	0.001	0.83
Eating duration	50 (25; 88)	100 (94; 100)	0.001	0.85
Eating desire	92 (58; 100)	100 (100; 100)	0.003	0.87
Fear of eating	94 (75; 100)	100 (100; 100)	0.001	0.84
Sleep	75 (50; 100)	88 (75; 100)	0.031	0.84
Fatigue	42 (25; 75)	92 (83; 100)	0.001	0.88
Communication	75 (63; 88)	100 (100; 100)	0.001	0.83
Mental health	95 (75; 100)	100 (100; 100)	0.001	0.88
Social functioning	100 (75; 100)	100 (100; 100)	0.001	0.94
Symptom score	66 (54; 86)	100 (95.5; 100)	0.001	0.94

^a^Mann–Whitney *U* test; significance level: *p* value < 0.05

^b^Cronbach’s *α* > 0.7

^c^Values are median and 25th;75th percentiles

### VFS measurements in the DM1 group

**Fig. 1:**
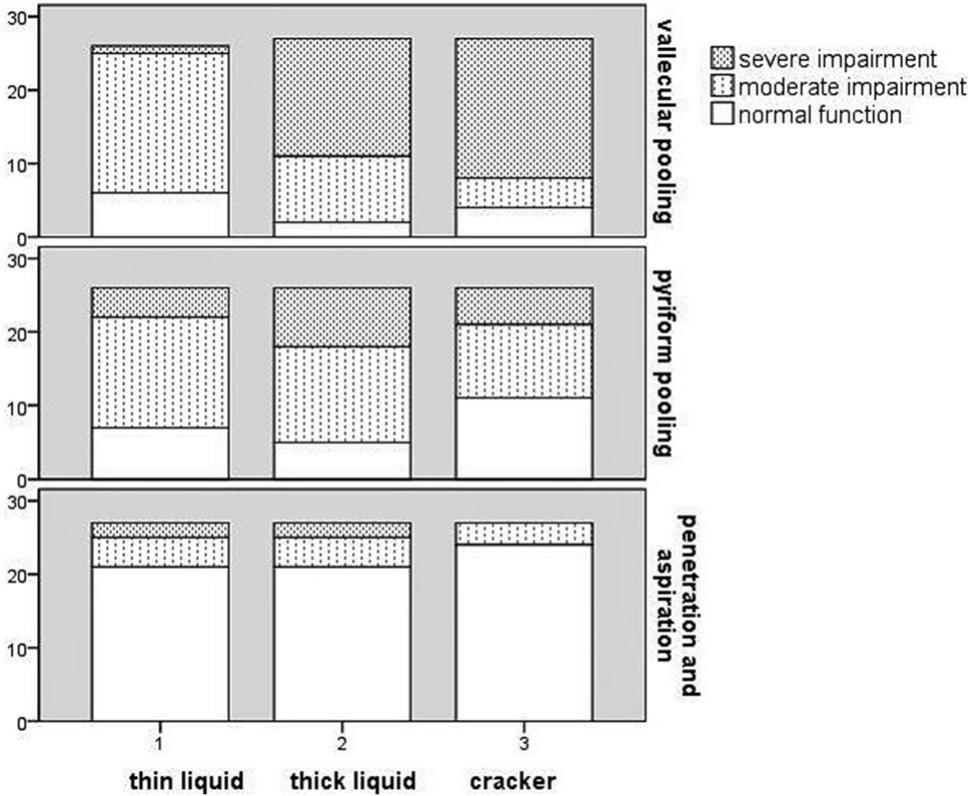
Frequency distribution of DM1 patients (*N* = 28) per consistency on three VFS outcome variables. The *y*-axis represents the frequency of occurrence in absolute numbers of patients. The *x*-axis indicates the consistency of each swallow. For the variables ‘vallecular and pyriform sinus pooling’, trace of pooling up to filling of less than 50% of the vallecular space/pyriform sinus is represented as moderate impairment. Filling of more than 50% of the vallecular space/pyriform sinus is represented as severe impairment. For the variable ‘penetration and aspiration’, penetration is represented as moderate impairment and aspiration as severe impairment

The VFS outcome measurements of the first subset of 28 DM1 patients were analyzed. Twenty of these patients were male and the mean age was 46.5 years. MIRS ranged from 1 to 5, median 5 (25th; 75th percentiles: 3.25; 5). Intra- and interrater agreement indices were substantial to almost perfect for all measured VFS variables. Analysis of the visuoperceptual variables revealed that swallowing function was impaired in the majority of the patients. The variable showing the highest level of impairment was postswallow vallecular pooling (Fig. 1). The percentages of patients with a score of two or more on the variable piecemeal deglutition were 46.1% (thin liquid), 48.1% (thick liquid), and 66.6% (cracker). One patient aspirated thin liquid and two patients aspirated both thin and thick liquid.

### Relationship between disease severity, SWAL-QOL, and VFS measurements in the DM1 group

The scores for the majority of the SWAL-QOL domains were lower in patients with proximal muscular weakness (MIRS 4 and 5). However, when comparing the score of each SWAL-QOL domain between the muscular impairment severity levels (MIRS 1 to 5), the differences did not reach statistical significance. Spearman’s correlation coefficient between severity of muscular impairment (MIRS) and SWAL-QOL showed a weak correlation in four domains: ‘general burden’ (rho = – 0. 241), ‘eating duration’ (rho = – 0.287), ‘fear of eating’ (rho = – 0.275), and ‘symptom score’ (rho = – 0.322). Two VFS outcome measurements correlated with MIRS: piecemeal deglutition of bite-sized cracker (rho = 0.466) and postswallow pyriform sinus pooling of thick liquid (rho = 0.376). Although these correlations were significant in the preliminary statistical analyses, none of them reached the level of significance after FDR correction for multiple-testing.

### Relationship between VFS measurements and SWAL-QOL

The SWAL-QOL domains ‘general burden’, ‘eating duration’, ‘eating desire’, and ‘fear of eating’, showed moderate correlation with VFS outcome measurements, though inconclusive after FDR correction (Table 2).

**Table 2:** Correlation between SWAL-QOL domains and VFS outcomes

VFS	SWAL-QOL			
	Eating duration	Eating desire	Fear of eating	General burden
Thin liquid				
Postswallow vallecular pooling		0.484 *p* = 0.025 *p* = 0.123*		
Postswallow pyriform sinus pooling	– 0.438 *p* = 0.012 *p* = 0.193*			
Thick liquid				
Postswallow vallecular pooling			– 0.410 *p* = 0.034 *p* = 0.180*	
Postswallow pyriform sinus pooling		– 0.453 *p* = 0.020 *p* = 0.228*		
Cracker				
Postswallow pyriform sinus pooling				0.532 *p* = 0.005 *p* = 0.064*
Piecemeal deglutition	– 0.478 *p* = 0.012 *p* = 0.123*			

The level of significance (*p*) before and after(*) the correction for multiple tests is presented

## Discussion

This study describes swallowing-related QoL in DM1 patients and evaluates the correlation between patient-reported impact of dysphagia on QoL, instrumental assessment of the swallowing function, and disease severity. The differences in the SWAL-QOL scores between the healthy control group and DM1 patients were significant and are in concordance with previous studies reporting lower SWAL-QOL scores in dysphagic subjects [8, 11]. Conversely, correlations between VFS outcome measurements, on the one hand, and SWAL-QOL domains and disease severity, on the other, were inconclusive after multiple testing correction.

In the DM1 group, the SWAL-QOL domain with the lowest scores (representing more impairment) was ‘fatigue’, followed by ‘eating duration’, and ‘symptom score’. The high frequency of fatigue found in this study is not surprising and is consistent with results from other studies [2, 12, 13]. In the domain ‘eating duration’, lower scores representing a longer amount of time to eat a meal were frequently found. A longer duration may be linked to weakness of the oral and pharyngeal muscles in DM1 patients [14]. Moreover, weak oropharyngeal muscles can impair the propulsion of the bolus from the mouth to the esophagus. An ineffective bolus propulsion usually generates repeated swallows in a physiological attempt to clear the residue from the oral cavity and pharynx [15]. As a consequence, a frequent occurrence of repeated swallows or ‘piecemeal deglutition’ may extend the duration of the meal.

Disease severity has been considered one of the main factors of health-related QoL in patients with neuromuscular disease. The impact of the progression of the disease on various aspects of health-related QoL, other than swallowing, has already been reported [5, 6, 16]. Our results showed a weak relationship between the severity of muscular impairment and SWAL-QOL domains as well as the severity of swallowing impairment. The hypothesis that the severity of the muscular impairment would have an impact on the SWAL-QOL domains should be further investigated in a longitudinal study.

This study analyzed a correlation between SWAL-QOL domains and VFS outcomes measurements to ascertain whether patients’ perception of the impact of dysphagia on QoL directly reflects the severity of the swallowing impairment. The moderate correlation between these two assessment approaches, lacking statistical significance after adjustment for multiple testing, suggests that patients with an impaired swallowing function not always report a decrease in their swallow-related QoL. Besides instrumental swallowing assessment using VFS the intrinsic characteristics of a tool that evaluated the impact of swallowing impairment on QoL and the variety of aspects involved in the eating process should also be considered. SWAL-QOL scores are based on patients’ personal perspective on the impact of dysphagia and are not a direct reflection of their physiological swallowing ability. Social, behavioral, and psychological aspects are entangled when feeding is self-assessed, and patients have different levels of perception and tolerance when facing a physical limitation. Especially in a disease with a slow progression such as DM1, patients adapt their actions and behavior, so their attitude can influence the scores on SWAL-QOL. In that light, our study confirms in the DM1 population the findings from a previous study that investigated a broad variety of dysphagic patients: SWAL-QoL and VFS measure different aspects or dimensions of the swallowing function, thus providing complementary information [17]. Moreover, it reinforces the importance of a multidimensional swallowing assessment, including an instrumental examination, such as fiberoptic endoscopic evaluation of swallowing (FEES) and/or VFS, in all DM1 patient with dysphagia.

SWAL-QOL provides valuable information on how these patients perceive and cope with their swallowing disability. This information is essential for rehabilitation programs, as it can guide the clinicians’ approach when proposing changes in food consistencies or an exercise program. Knowing which QoL aspects are impacted by dysphagia allow clinicians to look beyond the swallowing function, focusing on interventions that would have an effect on QoL. For instance, as fatigue and duration of the meals were SWAL-QOL domains affected by dysphagia in DM1 patients, the rehabilitation plan should include strategies to shorten the time patients spend eating a meal. Strategies such as, bolus modification, changes in food portion sizes, frequency of meals, etc. The success of a rehabilitation program is dependent on patients’ commitment, and changes in eating behavior are only possible if patients recognize the severity and the limitations imposed by their swallowing problem.

### Methodological limitations

The present study has some methodological limitations that should be taken into consideration. Though the DM1 patients scored significantly lower than the control group and all patients reported one or more dysphagia symptoms, most SWAL-QOL domains were not scored as severely impaired. The homogeneity of the SWAL-QOL scores, especially those with ceiling effects, is bound to have attenuated statistical correlations due to the restriction of range effect.

## Conclusion

SWAL-QOL provides information on how DM1 patients perceive their swallowing impairment and on the impact of dysphagia on their swallow-related QoL. There was no correlation between the severity of muscular impairment on one hand and VFS outcome and SWAL-QOL scores on the other. Interestingly, the weak to moderate, but eventually inconclusive correlations between VFS and SWAL-QOL outcomes suggests that these two tools measure different aspects of dysphagia in DM1 patients. If that is indeed the case, SWAL-QOL should not be taken as an indicator of the severity of dysphagia for these patients. However, SWAL-QOL should be part of the swallowing assessment as it provides complimentary information that could improve dysphagia management.
